# The Treatment of Subacute Bacterial Endocarditis with Penicillin

**Published:** 1947

**Authors:** C. Bruce Perry

**Affiliations:** Professor of Medicine in the University of Bristol


### THE TREATMENT OF SUBACUTE BACTERIAL ENDOCARDITIS WITH PENICILLIN

BY

C. BRUCE PERRY, M.D., Ch.B., F.R.C.P.

*Professor of Medicine in the University of Bristol.*

---

UP to 1945 reports of the treatment of bacterial endocarditis with penicillin were disappointing. Early in that year, however, more encouraging results were obtained with larger doses, and it became urgently necessary to determine the optimum dosage. With this end in view a committee was set up by the Penicillin Clinical Trials Committee with Professor R. V. Christie as secretary and representatives from fourteen centres. Various methods of treatment were tried at the different centres and the results pooled. As a result, by the end of September, 1945, 147 patients had been treated, and an optimum dosage determined. It was found that 0·5 mega units daily, by three-hourly injections, for twenty-eight days, gave the most successful results. It is to be hoped that further work will prove that less frequent administration may be possible by the use of penicillin in oil along the lines indicated by Sutton (1947). The results of the first 147 cases have been published (Christie, 1946). This paper is a brief record of the cases treated in Bristol. Thirteen of these were included in Christie's report.

In all, twenty-seven cases have been treated. In twenty-four, the infection was superimposed on previous rheumatic heart disease, and in three on a congenital defect. Fourteen have died. Of these, six died before completing the course, two relapsed after apparent arrest and died of a cerebral embolus, one died from infection from bed-sores with the endocarditis apparently arrested, and five died of cardiac failure seven days to three months after completing treatment and the arrest of the infection.

Of the thirteen survivors, eleven are leading the same lives as before the onset of the disease, and are back at work. One of these relapsed, but was cured by a second course. One had to have a leg amputated on account of a mycotic aneurysm, but is otherwise well, and the other has developed a relapsing rheumatic carditis and is still in hospital with this.

Thus nearly 50 per cent. of the cases treated have recovered from their infection. It must be noted that many of the cases treated had been ill for months before admission to hospital, and six died before the treatment could be completed. Before the introduction of penicillin no treatment was of any avail for bacterial endocarditis, but these results indicate the present importance of early diagnosis and prompt treatment if the patient is to be given a real chance of recovery.

REFERENCES.

[1] Sutton, F., *Clinical Journal,* 1947. lccvi, p. 8.
[2] Christie, R. V., *British Medical Journal,* 1945. 1, p. 381.

